# Hepatic parenchyma and vascular blood flow changes after TIPS with spectral CT iodine density in HBV-related liver cirrhosis

**DOI:** 10.1038/s41598-021-89764-6

**Published:** 2021-05-18

**Authors:** Lei Wang, Rengui Wang, Chunyan Zhang, Zhendong Yue, Hongwei Zhao, Zhenhua Fan, Yifan Wu, Yu Zhang, Fuquan Liu, Jian Dong

**Affiliations:** 1 Department of Interventional Radiology, Beijing Shijitan Hospital, Capital Medical University, No. 10 Tieyi St, Haidian District, Beijing, 100038 China; 2 Department of Radiology, Beijing Shijitan Hospital, Capital Medical University, No. 10 Tieyi St, Haidian District, Beijing, 100038 China

**Keywords:** Gastrointestinal diseases, Infectious diseases

## Abstract

To compare changes in spectral CT iodine densities of hepatic parenchyma and vessels before and after transjugular intrahepatic portosystemic shunt (TIPS) in hepatitis B virus (HBV)-related liver cirrhosis. Twenty-five patients with HBV-related liver cirrhosis who received TIPS for gastroesophageal varices bleeding were recruited. Each patient underwent three phases contrast CT before and after TIPS within 4 weeks, with the raw data reconstructed at 1.25-mm-thick slices. Iodine density (in milligrams per milliliter) was measured on iodine-based material decomposition image. Multiple regions of interest (ROIs) in liver parenchyma, aorta and portal vein were selected from three slices of images. Portal vein trunk was set as the central one, and mean liver parenchymal iodine densities from arterial phase (AP), venous phase (VP) and equilibrium phase (EP) were recorded. Quantitative indices of iodine density (ID), including normalized ID in liver parenchyma for arterial phase (NIDLAP), ID of liver parenchyma for venous phase (IDLVP), ID of portal vein in venous phase (IDPVP) and liver arterial iodine density fraction (AIF), were measured and compared before and after TIPS. Based on Child–Pugh stage, 4, 12 and 9 patients were classified as grade A, B, and C, respectively. Liver volume was comparable before and after TIPS (1110.5 ± 287.4 vs. 1092.0 ± 276.3, *P* = 0.28). After TIPS, ID was decreased in aorta (146.0 ± 34.5 vs. 120.9 ± 30.7, *P* < 0.01) whereas increased in liver parenchyma at arterial phase, as demonstrated by IDAP (9.3 ± 3.1 vs. 13.4 ± 4.4 mg/mL) and AIF (0.40 ± 0.11 vs. 0.58 ± 0.11, *P* < 0.01). For venous or equilibrium phase, quantitative indices remained stable (23.1 ± 4.5 vs. 23.0 ± 5.3, 19.8 ± 4.1 vs. 19.4 ± 4.6) mg/mL (Ps > 0.05). For portal vein, ID and NID were increased after TIPS (23.1 ± 11.7 vs. 36.5 ± 13.0, 16.4 ± 8.5 vs. 31.8 ± 12.8) (P < 0.01). No positive correlation between iodine density and preoperative Child–Pugh score was observed. Based on iodine density measurement, spectral CT as a noninvasive imaging modality may assess hepatic parenchyma and vascular blood flow changes before and after TIPS in HBV-related liver cirrhosis.

**Clinical registration number**: ChiCTR- DDC-16009986.

## Introduction

Hepatitis B virus (HBV) infection is the leading cause of liver cirrhosis in Asian countries^1–3^. One-year mortality of cirrhosis varies from 1 to 57%, depending on clinical decompensation and serious adverse events, such as gastroesophageal varices bleeding^2,3^. Compared with conservative therapy and surgery, TIPS was reported to exert better therapeutic effects and to reduce mortality for acute variceal bleeding in liver cirrhosis, especially at Child B or C stage^4–7^. However, blood supply could be decreased in liver parenchyma after TIPS since portal vein blood partially flows directly to inferior vena cava through portal-systemic shunt, which could cause increased risk of hepatic encephalopathy and liver failure^5,8,9^. Therefore, it is important to quantify decrease in blood supply, in an attempt to predict development of complications and to improve prognosis.

Noninvasive imaging modalities have been applied in assessment of hepatic blood flow changes in liver lesions, including perfusion CT and MR^4,9^. Notably, blood perfusion in liver parenchyma might be decreased significantly after TIPS^9^. However, radiation dose of CT perfusion was very high, and accuracy of MR perfusion would be affected by metal coil artifacts after TIPS with gastric coronary vein embolization. Recently, spectral CT has been applied as a quantitative imaging tool in liver lesions, such as hemangioma and hepatocellular carcinomas, with increased sensitivity for differential diagnosis^10–12^. Furthermore, Based on iodine density from material decomposition, spectral CT exhibits capability in quantifying liver fat concentration and staging in liver cirrhosis^13,14^. Therefore, our purpose is to investigate potential feasibility of spectral CT iodine density as a non-invasive imaging modality in assessment of hepatic blood flow changes after TIPS in patients with HBV-related liver cirrhosis.

## Materials and methods

### Patients

This study has been performed in accordance with Declaration of Helsinki, and approved by institutional ethics committee in our hospital (Beijing Shijitan hospital, Capital Medical University) complying with Ethical Principles for Medical Research Involving Human subjects, and registered in Clinical Trial Registry with number of ChiCTR-DDC-16009986. All the examinations Informed consent was signed by each patient. From January to May in 2019, all patients with gastroesophageal bleeding resulted from hepatitis B were treated with TIPS. The inclusion criteria were as follows: (1) patients with HBV-related liver cirrhosis, (2) Contrast CT was performed within 4 weeks before and after TIPS, and (3) Child–Pugh score was evaluated within 2 weeks before TIPS. Exclusive criteria were as follows: (1) patients with malignant hepatic tumors, either primary or metastatic, (2) any conditions affecting liver blood flows, including iatrogenic (liver surgery, splenectomy, and TIPS) or portal vein lesions (portal venous thrombosis and portal cavernous transformation), (3) patients with allergy to iodinated contrast media, (4) estimated glomerular filtration rate (GFR) lower than 30 mL/min, and (5) severe motion artifacts. The complications, such as hepatic encephalopathy, coma, and liver failure, were recorded during 4-week follow-up.

### Spectral CT examination and quantitative indices measurement

Quadruple-phase (pre-contrast, arterial, venous and equilibrium phase) contrast enhanced CT (Revolution, GE Healthcare, WI) was performed^14–16^. All patients were scanned with spectral imaging mode with scanning parameters as follows: fast switch tube voltage 80/140 kVp, automatic tube current from 100 to 600 mA with noise index set as 9, 8 cm detector, slice thickness of 5 mm, rotation speed of 0.5 s, helical pitch of 0.992:1, and 40% Asir. Nonionic contrast media (Omnipaque 350) were injected through antecubital vein at a rate of 5 mL/sec, with a total volume of 80–120 mL (1.5 mL per kilogram of body weight). Hepatic arterial phase (AP) imaging was determined by automatic scan triggering software (SmartPrep; Revolution CT, GE Healthcare, WI, https://www.gehealthcare.com/) when trigger attenuation threshold (120 HU) reached the level of supraceliac abdominal aorta, while portal venous and equilibrium phase was initiated at 45 and 120 s after AP phase, respectively. All raw data were reconstructed with 2.5 -mm-thick slices. Then, monochromatic images at 70 keV, water- and iodine-based material decomposition images were analyzed. All post-processions were performed in Advanced Workstation (Version 4.7, GE Healthcare, WI, https://www.gehealthcare.com/).

#### Iodine density measurement

**Figure 1:**
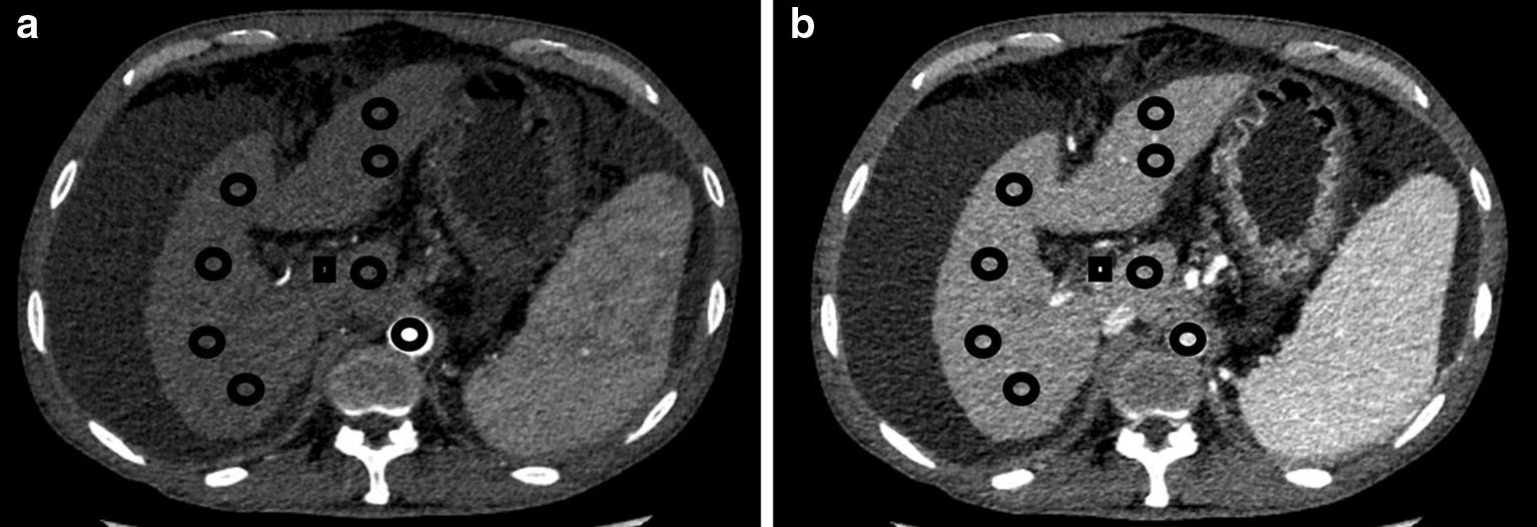
Regions of interest (ROIs) measurement of quantitative indices. Multiple ROIs were placed in liver parenchyma (circle ROI), aorta (circle ROI) and portal vein (square ROI) in arterial phase (**1a**) and venous phase (**1b**), with average area about 100 mm^2^, respectively. The averages were calculated as the iodine density.

Iodine densities (in milligrams per milliliter) were measured on iodine-based material decomposition images, including non-contrast, AP and PVP phases. Multiple regions of interest (ROI) (mean area larger than 100) were placed in liver parenchyma from different hepatic lobes, including lateral, medial, anterior, and posterior segments at the level of hepatic hilum, with large vessels, liver cyst, calcification and prominent artifacts to be avoided carefully. All ROIs were placed at 3 different levels, with hepatic hilar serving as the central one. Furthermore, size, shape, and position of ROIs were kept consistent among images by applying copy-and-paste function. Then, an average value was calculated as iodine density (ID)^15^. Quantitative indices of ID were measured according to Dong’s report^15^ as follows: (1) ID of liver parenchyma at arterial phase (ID_LAP_) or venous phase (ID_LVP_) was calculated as the difference between AP or VP and non-contrast phase, respectively. (2) ID of aorta in AP (ID_AO_) and ID of portal vein in VP (ID_PVP_) were recorded. (3) Normalized ID was defined as NID_LAP_ = ID_LAP_/ID_AO._ (4) Liver arterial iodine density fraction (AIF) was defined as: AIF = ID_LAP_/ID_LVP_ (Fig. 1).

#### Liver volume measurement

**Figure 2:**
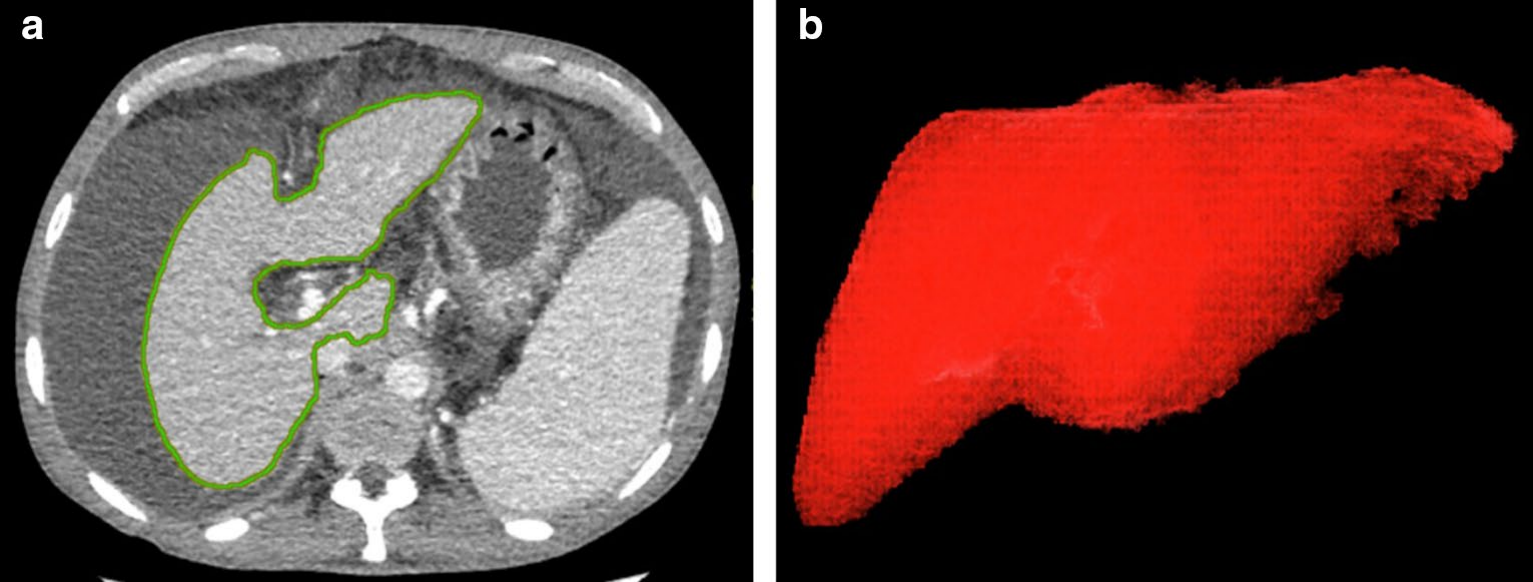
Liver volume was measured on images of enhanced CT at venous phase (**2a**), with software (AW 4.7, total liver and segment separation), and finally the whole liver volume would be calculated automatically and shown with volume rendering (**2b**).

Liver volume was measured on images of enhanced CT at venous phase^17,18^. First, the images of contrast CT venous phase were analyzed by software named as total liver and segment separation in Advanced Workstation (Version 4.7, GE Healthcare, WI, https://www.gehealthcare.com/), then “activate AutoSelect tool” was applied to adjust the edge of the liver by radiologists slice by slice, and finally the liver volume would be calculated automatically (Fig. 2).

### Statistical analyses

Statistical analysis was carried out using SPSS 22.0 software (SPSS, Inc., Chicago, Illinois, USA, https://www.ibm.com/products/spss-statistics). Pair-sample *t*-tests were used to compare quantitative indices before and after TIPS. Pearson correlation analyses were performed to assess associations between Child–Pugh scores and quantitative indices before TIPS. A *P* < 0.05 was set as statistical significance.

## Results

Totally, 25 patients (F/M, 7/18, age range, 29–74 years) were enrolled in our study. Based on Child–Pugh stage, 4, 12 and 9 patients were classified as grade A, B and C, respectively, before TIPS. Six patients were treated with TIPS only, whereas 21 with TIPS combined gastric coronary vein embolization. Two patients developed hepatic encephalopathy within 2 weeks after surgery, and recovered in 4-week follow-up with conservative therapy. No hepatic coma or liver failure occurred.

Quantitative indices from spectral CT were compared before and after TIPS. Liver volume remained stable before and after TIPS (1110.5 ± 287.4 vs. 1092.0 ± 276.3, *P* = 0.28). ID in liver parenchyma, NIDL_AP_ and AIF were increased after TIPS. By contrast, ID in liver parenchyma at venous or equilibrium phase was stable after TIPS. ID in aorta was decreased after TIPS. For portal vein, ID and NIDPV_AP_ were increased, while ID at venous or equilibrium phase was stable after TIPS (Table 1). No positive correlation of iodine density with preoperative Child–Pugh score was observed.

**Table 1:** Comparisons of Iodine density before and after TIPS.

Iodine density (mg/ml)	Before	After	*P*
Liver parenchyma			
AP	9.3 ± 3.1	13.4 ± 4.4	< 0.01
VP	23.1 ± 4.5	23.0 ± 5.3	0.88
EP	19.8 ± 4.1	19.4 ± 4.6	0.57
NID_LAP_ (*10^–2^)	6.5 ± 2.4	11.1 ± 2.5	< 0.01
AIF	0.40 ± 0.11	0.58 ± 0.11	< 0.01
Aorta_AP_	146.0 ± 34.5	120.9 ± 30.7	< 0.01
Portal Vein_AP_	23.1 ± 11.7	36.5 ± 13.0	< 0.01
Portal Vein_VP_	55.5 ± 9.1	53.0 ± 10.8	0.17
NIDPV_AP_ (*10^–2^)	16.4 ± 8.5	31.8 ± 12.8	< 0.01
Liver volume	1110.5 ± 287.4	1092.0 ± 276.3	0.28

Data are represented as mean ± standard deviation. *AP* arterial phase, *VP* venous phase, *EP *equilibrium phase, *AIF* liver arterial iodine density fraction, *NID*_*LAP*_ normalized iodine density of liver parenchyma in arterial phase, *NIDPV*_*AP*_ normalized iodine density of portal vein in arterial phase.

## Discussion

TIPS is an effective therapy for gastroesophageal varices bleeding caused by portal hypertension in liver cirrhosis^1,5–7^. Once shunts between portal vein and inferior vena cava are established, portal hypertension would be alleviated, so that the risk of gastroesophageal varices bleeding would be reduced^5,6^. However, portosystemic shunt after TIPS can cause further reduction in hepatic blood flow, which impairs hepatic detoxification, and thus induces complications such as hepatic encephalopathy and liver failure^2^. In our study, ID of liver parenchyma or portal vein at arterial phase would obviously increase, whereas peak value of aorta would decrease. By contrast, ID in liver parenchyma or portal vein remains stable at venous or equilibrium phase after TIPS. Therefore, ID measured on spectral CT has great potential for non-invasive quantitative evaluation of liver blood flow changes after TIPS.

Spectral CT has been reported as a noninvasive tool for quantitative assessment of liver fibrosis^14–16^. In our study, ID_LAP_, NID_LAP_ and AIF were increased significantly after TIPS, suggesting that arterial blood supply would be increased in liver parenchyma after TIPS. The liver is a solid organ with dual blood supply, i.e., hepatic artery and portal vein. Under normal circumstances, hepatic artery accounts for about 25% of total blood supply. While in patients with liver cirrhosis, the proportion of arterial blood supply is increased significantly (0.40 ± 0.11), which is consistent with an increased AIF as reported by Dong et al.^15^. However, after TIPS, shunts can cause partial portal vein blood to flow directly back into systemic circulation, so that portal vein blood supply to liver parenchyma is further reduced. With increased arterial and decreased portal vein blood supply, AIF would be further increased after TIPS (0.40 ± 0.11 vs. 0.58 ± 0.11, *P* < 0.01). Therefore, dynamic changes in hepatic blood flows at arterial phase can be evaluated by spectral CT iodine density in hepatic parenchyma as a noninvasive assessment.

In our study, no statistical difference in iodine density was observed between portal vein and equilibrium phases in liver parenchyma after TIPS, which is inconsistent with previous reports, where blood supply of liver parenchyma was decreased significantly with CT perfusion^8,9^. This inconsistency may be related to abnormal hepatic blood flow and pharmacokinetics of contrast medium in liver cirrhosis. Because iodine density was calculated at venous or equilibrium phase set at 70 s and 120 s, respectively, and it only reflects static distribution of contrast media in liver parenchyma at that moment. Due to the distorted structures of pseudo-lobules in HBV-related liver cirrhosis, normal wash-in and wash-out of contrast media would be disturbed. Besides, common vascular distortion includes arteria-portal fistula, arteriovenous fistula, and abnormal perfusion of liver, could also be detected in liver cirrhosis. Thus, it is impossible to quantify actual blood supply in liver parenchyma from portal vein perfusion with iodine density at venous or equilibrium phase at each time point. Further research, such as CT and MR perfusion for specific quantitative analysis, is needed to evaluate actual changes in liver blood supply^4,9^.

Dynamic changes in ID exhibited different trends in hepatic vascular system on three-phase contrast CT. In our study, ID was decreased in aorta (146.0 ± 34.5 vs. 120.9 ± 30.7, *P* < 0.01), whereas increased in portal vein (23.1 ± 11.7 vs. 36.5 ± 13.0, *P* < 0.01) or NIDPV_AP_ (16.4 ± 8.5 vs. 31.8 ± 12.8, *P* < 0.01) at arterial phase. By contrast, ID in portal vein at venous phase remained stable (55.5 ± 9.1 vs. 53.0 ± 10.8, *P* = 0.17). Thus, both portal and systematic circulation would have been affected after TIPS, especially at arterial phase. Portal-systemic shunts would result in hepatic blood flow and pharmacokinetic changes in contrast media, however, biopathology for this consequence needs further investigation.

In our study, spectral CT ID in liver parenchyma or blood vessels shows no positive correlation with preoperative Child–Pugh score, which is inconsistent with previous reports^15^. This may be related to adoption of Child–Pugh score instead of Child–Pugh grading. Besides, liver volume (1110.5 ± 287.4 vs.1092.0 ± 276.3, *P* = 0.28) was stable after TIPS. However, 2 patients developed hepatic encephalopathy within 2 weeks after TIPS. Interestingly, for them, liver volume was decreased by more than 10 percent, whereas blood ammonia was increased, as potential indication for quantitative assessment of TIPS-related complications.

There are some limitations in our study. Firstly, contrast enhanced CT is performed at three time points, including arterial, venous and equilibrium phases, however, ID only reflects static snapshot of blood distribution in liver parenchyma at a specific time point, rather than actual blood perfusion. So, ID is indirect reflection of blood supply in liver parenchyma, and more studies should be performed to quantify actual blood perfusion, especially in comparison of CT and MR perfusion for liver blood flow. Secondly, our study has been focused on HBV-related cirrhosis, whether iodine density could be used in other diseases, such as hepatitis C, alcoholic hepatitis and autoimmune hepatitis, needs further investigation. Thirdly, ID shows no correlation with Child–Pugh score in our study, which is inconsistent with Dong’s report. This may be related to the small sample size, so studies in larger patient population should be performed in the future.

In conclusion, spectral CT iodine density demonstrates increased blood supply in liver parenchyma and portal vein at arterial phase after TIPS, which has potential capability to evaluate hepatic blood flow changes in HBV-related portal hypertension and liver cirrhosis as a non-invasive quantitative imaging modality.
